# Individuality and Stability in Male Songs of Cao Vit Gibbons (*Nomascus nasutus*) with Potential to Monitor Population Dynamics

**DOI:** 10.1371/journal.pone.0096317

**Published:** 2014-05-02

**Authors:** Jun-Juan Feng, Liang-Wei Cui, Chang-Yong Ma, Han-Lan Fei, Peng-Fei Fan

**Affiliations:** 1 Institute of Eastern-Himalaya Biodiversity Research, Dali University, Dali, Yunnan, P. R. China; 2 College of Life Science, Southwest Forestry University, Kunming, Yunnan, P. R. China; 3 Key Laboratory of Forest Disaster Warning and Control in Yunnan Province, Southwest Forestry University, Kunming, Yunnan, P. R. China; Utrecht University, Netherlands

## Abstract

Vocal individuality and stability has been used to conduct population surveys, monitor population dynamics, and detect dispersal patterns in avian studies. To our knowledge, it has never been used in these kinds of studies among primates. The cao vit gibbon is a critically endangered species with only one small population living in a karst forest along China-Vietnam border. Due to the difficult karst terrain, an international border, long life history, and similarity in male morphology, detailed monitoring of population dynamics and dispersal patterns are not possible using traditional observation methods. In this paper, we test individuality and stability in male songs of cao vit gibbons. We then discuss the possibility of using vocal individuality for population surveys and monitoring population dynamics and dispersal patterns. Significant individuality of vocalization was detected in all 9 males, and the correct rate of individual identification yielded by discriminant function analysis using a subset of variables was satisfactory (>90%). Vocal stability over 2–6 years was also documented in 4 males. Several characters of cao vit gibbons allowed long-term population monitoring using vocal recordings in both China and Vietnam: 1) regular loud calls, 2) strong individuality and stability in male songs, 3) stable territories, and 4) long male tenure. During the course of this research, we also observed one male replacement (confirmed by vocal analysis). This time- and labor-saving method might be the most effective way to detect dispersal patterns in this transboundary population.

## Introduction

Vocal communication is very common in birds, primates, and cetaceans that are living in dense forest or under water where olfactory and visual signals are limited or not feasible. For signal receivers, identification of the caller serves both to discriminate members from intruders and to maintain affiliative relationships with other individuals [1]. Signalers are expected to actively broadcast their identity with distinctive cues provided there is a benefit to being accurately identified [2]. Accordingly, vocal individuality has been demonstrated widely among different animal taxa [2]–[6], and it has also been used to evaluate territorial boundaries, map home ranges, conduct population surveys, monitor population dynamics, and detect dispersal patterns [7]–[11]. However, long-term stability of vocal individuality is a prerequisite for its utilization in these techniques [9].

Gibbons are small, arboreal, territorial apes, inhabiting the broad-leaved evergreen forests of Southeast Asia. They typically live in small groups comprised of one adult pair and 2–3 offspring [12]. All gibbons produce species-specific and sex-specific songs lasting 10–30 min in the early morning, and paired mates often combine their respective vocalizations into well-coordinated duets (with the exceptions of *Hylobates klossii* and *H. moloch*) [13], [14]. Several functional explanations of gibbon songs in which selection may have favored acoustic individuality and individual recognition have been proposed; these include regulating spacing among groups, defense of resources, mate attraction, and strengthening or advertising the pair bond [1], [3], [13], [15]–[17]. Although acoustic individuality has already been reported in several gibbon species: *Hylobates agilis* [1], [3], *H. klossii* [18], *H. moloch* [19], [20] and *N. concolor* [21], the vocal stability of gibbon songs has seldom been assessed (but see Fan et al. [22]).

In this study, we tested the vocal individuality and stability of male songs from cao vit gibbons (*Nomascus nasutus*) over the course of 2–6 years. Here we discuss the possibility of using vocal individuality to monitor the population dynamics of the species. Cao vit gibbons, also known as eastern black crested gibbons, are a Critically Endangered species [23] and listed as one of the ‘World's 25 Most Endangered Primates’ [24]. There is only one population remaining, with about 110 individuals surviving in a karst forest patch crossing the China-Vietnam border [25]. Accurate population estimates and monitoring are essential for the conservation of the species; however, the karst forest in which they live hampers close following and habituation. Researchers are rarely able to observe them from distances of less than 50 m [22], [26]. Adult females could be easily distinguish based on different shapes of their obvious white face ring and large black crown patch, but the adult males are almost entirely black without obvious distinguishing marks [27], making males difficult to identify easily in the forest. Capturing and handling these endangered gibbons safely would be practically impossible and would not likely be allowed by authorities. The international border splitting their habitat makes it difficult to monitor the whole population, as it is not possible to follow individuals that disperse across the border. Therefore, a time- and labour-saving method that could be used easily in both countries is needed to conserve the species. Using vocal individuality would be the most effective way to identify individuals in this situation [9].

## Methods

### Ethics Statement

No animals were handled during this study. Research permissions were issued by Mr. Tan Wu-Jing, the director of Bangliang Nature Reserve, and Mr. Nong Van Tao, the director of Cao Vit Gibbon Conservation Area. All work was done in accordance with guidelines of the national anthorities of both China and Vietnam.

### Study Sites and Subject Animals

We carried out this study at two sites that form a contiguous zone across the border of China and Vietnam [28]: Bangliang Nature Reserve (22°55′N, 106°29′-30′E), Jingxi County, Guangxi, China and The Cao Vit Gibbon Conservation Area (22°54′-55′N, 106°31′-32′E), Trung Khanh, Cao Bang, Vietnam. The area is dominated by tropical monsoon forest at the center of the karst limestone block, and surrounded by degraded secondary forest and scrub [28].

Since 2008, only three gibbon groups have lived in Bangliang Nature Reserve [29], all of which were chosen for this study. G1 consisted of an adult male (G1 male) and a juvenile male in 2007. The juvenile male started to sing solo bouts in 2011 and dispersed in August 2013. We named it G1 subadult male in this study. A new male (G1 new male) replaced the G1 male between May and September 2012. G2 consisted of only one adult male and no juvenile or subadult males. G4 also consisted of an adult male (G4 male) and a juvenile male in 2007. The juvenile male developed into a subadult (G4 subadult male) and produced solo bouts in 2011, then dispersed in 2012. In total, we recorded solo or duet bouts from six different males in these three groups all of whom have been identified by morphological and behavioral characters (G1 male, G1 new male, G1 subadult male, G2 male, G4 male and G4 subadult male) (Table 1). Each of these three study groups has been intensively studied since 2007 and their home ranges are well known [26], [29]–[31].

**Table 1:** Sample size for 9 male cao vit gibbons in this study.

Individuals	Number of male phrases/(Number of song bouts)					Total
	2007	2008	2009	2011	2012	
G1 male	8/(1)	42/(7)	4/(1)	11/(5)	17/(3)	82/(17)
G1 new male					66/(16)	66/(16)
G1 subadult male				15/(2)	85/(14)	100/(16)
G2 male	10/(2)	22/(3)	38/(5)	56/(14)	8/(3)	134/(27)
G4 male			6/(1)	54/(6)	30/(5)	90/(12)
G4 subadult male				22/(3)		22/(3)
V1				30/(6)		30/(6)
V2				11/(3)		11/(3)
V3				46/(10)		46/(10)
Total	18/(3)	64/(10)	48/(7)	245(49)	206/(41)	581/(110)

We also visited The Cao Vit Gibbon Conservation Area in Vietnam in June 2011. Although there were more than 10 groups living in this area, we were not permitted to observe every group and only recorded clear song bouts from three groups near a ranger station. Each group had one adult male (V1, V2, and V3 in Table 1). Vietnamese rangers in the Cao Vit Gibbon Conservation Area have monitored these three study groups for several years, so their home ranges are partially known.

### Vocal Recording

We recorded song bouts in Bangliang from September 2007 to October 2012, excluding 2010 and in Trung Khanh during June 2011. Given that these gibbons normally produce song bouts in the early morning [32], we occupied the listening posts from 0600–1200 h. We recorded gibbon song bouts using a Sony TC-D5 Pro2 recorder with a Sony C-76 directional microphone and Sony analogue tapes (Sony C-90EFB and Sony C-60EFB).

### Acoustic Analysis

All vocal recordings were digitized with a sample rate of 44.1 kHz and a sample size of 16 bits using the Sony TC-D5 Pro2 recorder and a 50 kHz Analog I/O Card (Engineer Design). The time-frequency sonograms and measurements of vocalizations were carried out using Signal/RTS 4.0 (Engineer Design). The FFT size of the sonograms was 1024 points with a frequency resolution of 43.1 Hz and a time resolution of 23.2 ms. Acoustic terminology used in this research (Table 2) follows that proposed by Konrad and Geissmann [33], Ruppell [34] and Feng et al. [35].

**Table 2:** Acoustic terms and definitions of gibbon song (following Konrad and Geissmann [33], Ruppell [34] and Feng et al. [35]).

Term	Definition
Note	A single continuous sound of distinct frequency or frequency modulation that may be produced during either inhalation or exhalation
Roll	A characteristic of notes produced by the male wherein each roll includes a steep increase in frequency followed by a steep decrease
Phrase	A single vocal activity consisting of a larger or looser collection of notes or elements or both that may be produced together or separately
Male sequence	A complete sequence produced by the male, is made up of boom, aa note, pre-modulated note and modulated figure. The female dose not sing during this period
Song bout	All song notes of a gibbon group with periods of silence of <10 min

**Figure 1:**
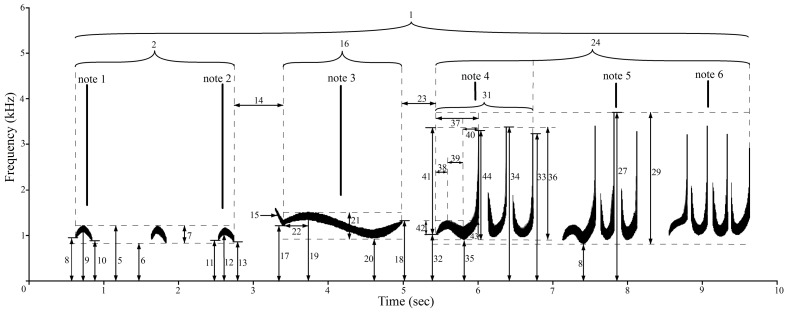
Sonogram (only fundamental frequencies) of a fully male phrase showing most of the variables based on the measurements.

Recordings for which the sound quality was poor or the caller was uncertain were excluded. Male cao vit gibbons produced complex and differently structured phrases [35]. Male phrases responding to female great calls were excluded, because they are much more complex and differently structured from male phrases when females do not produce great calls, making it difficult to measure them using the same method [35]. We measured one of every six male phrases with five unmeasured phrases between two measured samples to increase independence between phrases. We measured the time and frequency of every inflection point of the fundamental frequencies of the male phrase including the first and last of the aa notes, pre-modulated notes and modulated figures (Fig. 1). We defined 44 variables based on the measurements to quantify the acoustic characteristics of the male phrase (Table 3). All measurements were done by Feng JJ to avoid interobserver differences.

**Table 3:** Descriptions of acoustic variables and variables selected by 6 DFA analyses (“+”  =  Selected).

No.	Part	Description	Individual differences			Acoustic stability		
			1st	2nd	3rd	2007–2008	2007–2009	2007–2011
1	Entire phrase	Total duration of entire phrase		+	+			+
2	aa notes	Total duration		+	+			
3		Number of notes	+		+			+
4		**Average duration of note1 and note2**	+	+	+			+
5		**Highest frequency**	+	+	+	+	+	+
6		Lowest frequency	+		+			
7		Frequency range		+			+	
8	Note 1	**Start frequency**	+	+	+			
9		Middle frequency	+	+				+
10		**End frequency**	+	+	+			
11	Note 2	Start frequency						
12		Middle frequency						
13		End frequency						
14		Time interval between note2 and note3	+			+		+
15	Note 3	Occurrence of descending at the start		+	+		+	+
16		**Duration**	+	+	+			+
17		Start frequency	+	+			+	
18		**End frequency**	+	+	+		+	+
19		Highest frequency	+					
20		**Lowest frequency**	+	+	+	+		+
21		Frequency range		+	+		+	+
22		**Duration from start to the highest frequency point**	+	+	+	+	+	+
23		Time interval between note3 and note4		+				
24	Modulated figures	Total duration						
25		**Number of notes**	+	+	+			+
26		**Average duration of every note**	+	+	+			+
27		Highest frequency						+
28		**Lowest frequency**	+	+	+		+	+
29		Frequency range		+		+	+	
30	Note 4	**Roll present or not**	+	+	+			+
31		**Duration**	+	+	+		+	+
32		**Start frequency**	+	+	+		+	+
33		End frequency		+				
34		Highest frequency			+			
35		**Lowest frequency**	+	+	+		+	+
36		Frequency range	+					
37	Initial part of Note 4	**Duration**	+	+	+			+
38		Duration of initial part	+					
39		Duration of middle part				+	+	+
40		**Duration of terminal part**	+	+	+	+	+	+
41		Frequency range of start to highest						
42		**Frequency range of initial part**	+	+	+		+	+
43		**Frequency range of middle part**	+	+	+	+	+	+
44		Frequency range of terminal part		+				

Variables repeatedly selected in all three individual difference analyses to test vocal individuality are bolded.

In total, we analyzed 581 phrases of 110 song bouts from 9 male individuals (Table 1); however, the sample size was not equal to 9 male individuals throughout 5 years, because of the absence of individuals in some years, and variable recording quality.

### Statistical Analysis

We used stepwise discriminant function analysis (DFA) to identify individual differences and acoustic stability [1], [21], [22], [33], [36]. We chose Wilks' lambda as the criterion for variable selection. To test the significance of change in the selection criterion when we entered or removed a variable from a model, the probability of F was used and p-to-enter  = 0.05 and p-to-remove  = 0.10 were applied as significance levels [21], [22], [33]. To avoid empty cells in the data matrix, the start, middle, and end frequency of note 2 (No. 11, 12 and 13 in Table 3) were excluded. Thus 41 variables were chosen for every DFA. We did not replace the missing values with the mean.

In order to demonstrate individuality among the 9 males, a split-sample method was used to classify the remaining individuals [1], [22]. We randomly selected approximately 50% of phrases to calculate the discriminant functions, which we then used to reclassify the remaining samples. We repeated this process three times. In order to examine acoustic stability over time, a cumulative method was used to identify individuals in future years [22], [37]. We used phrases recorded in former years to calculate DFA functions and then used them to discriminate phrases in the following year. For example, DFA functions were calculated from phrases recorded between 2007–2008 and were used to discriminate phrases recorded in 2009. All statistical analyses were carried out using SPSS 16.0.

## Results

### Vocal Individuality

**Figure 2:**
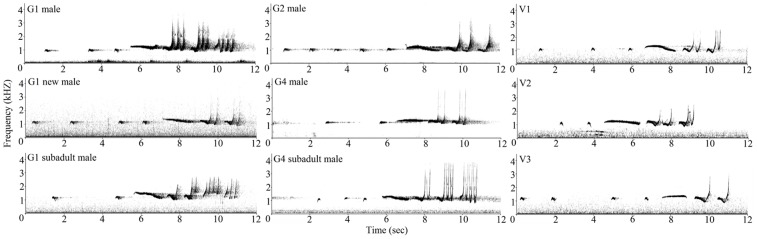
Typical sonograms of the 9 adult male cao vit gibbons in this study.

Of the 41 variables analyzed, 27, 30, and 26 variables were selected to classify phrases of individuals in 3 separate DFAs, and 19 variables were repeatedly selected by all 3 discriminant functions (Table 3). In each situation, DFA yielded a high correct rate of acoustic individual identification, even with the 19 repeatedly selected variables (Table 4). Typical sonograms of the 9 males are shown in Figure 2.

**Table 4:** The rate of correct individual recognition with different variables using DFA for male cao vit gibbons.

Individuals	Correct rate (%) (41 variables)			Correct rate (%) (19 variables)		
	1st	2nd	3rd	1st	2nd	3rd
G1 male	93.1	96.2	90.0	97.8	97.3	97.8
G1 new male	94.3	92.6	100	85.2	96.7	84.8
G1 subadult male	95.6	96.0	91.5	93.6	90.0	97.7
G2 male	98.4	100	100	97.3	98.6	98.6
G4 male	91.8	82.9	84.8	89.1	77.3	83.7
G4 subadult male	81.2	75.0	85.7	90.0	75.0	100
V1	100	100	100	100	100	100
V2	100	100	100	100	100	100
V3	95.8	95.0	100	96.7	96.3	96.3
Mean	94.7	94.2	94.0	94.4	92.9	94.5

### Vocal Stability

Vocal stability was documented in all 4 males over 2–6 years (Table 5). For G1 male, the identification accuracy rates in 2009 and 2011 were 100%. And the DFA easily identified the male replacement in 2012 with the accuracy rate decreased to 23.9%. G2 male's vocalization remained very stable during the study and the correct rate was 100% in all years. Although the accuracy of discrimination was low for G4 male in 2011 and G1 subadult male in 2012, the majority of phrases were predicted to be from the correct individuals (Table 6).

**Table 5:** DFA classification results for male phrases of cao vit gibbons between years. G1 male was replaced by the G1 new male in 2012.

Individuals	Discrimination				Discrimination				Discrimination			
	2007–2008		2009		2007–2009		2011		2007–2011		2012	
	n	%	n	%	n	%	n	%	n	%	n	%
G1 male*	50	100	4	100	54	100	11	100	51	100	46	23.9
G2 male	32	100	38	100	70	100	56	100	126	100	8	100
G4 male					6	100	54	51.9	59	100	30	100
G1 subadult male									15	100	80	77.5

**Table 6:** DFA classification results for male phrases of G4 male in 2011 and G1 subadult male in 2012.

Individuals	Predicted group membership							
	G1 male		G1 subadult male		G2 male		G4 male	
	n	%	n	%	n	%	n	%
G4 male	13	24.1			13	24.1	28	51.9
G1 subadult male			62	77.5	10	12.5	8	10.0

## Discussion

In this study, we provide the first demonstration of vocal individuality and stability in male songs of cao vit gibbons. Discriminant function analysis with 19 variables was able to classify all 9 males studied, and in most cases, male songs recorded from former years were able to identify individuals recorded in the following year. Due to the international border splitting the nature reserve and the very small gibbon population in China, we could record only 9 individuals to test vocal individuality and 4 individuals to test vocal stability over the 6 years' study. While we acknowledge the small sample size in this study, our results are valuable given the extraordinary difficulty of studying and conserving this critically endangered species. Given that the entire species is comprised of fewer than 20 predominantly inaccessible groups [25], identification of vocal individuality, although imperfect, will likely provide the only repeatable monitoring data for cao vit gibbons.

In two situations when we used phrases recorded from previous years to identify phrases in the following year, the identification accuracy was low. One possible explanation is that the number of phrases used to calculate the discriminant functions was much smaller than the number of phrases waiting for classification (G4 male in 2011 and G1 subadult male in 2012). Studies have shown that vocal features can vary with social context in birds [38], dolphins [39], and primates [40], [41]. Gibbons may also change their calls in response to differences in social context, such as visual contact with neighboring groups or losing contact with group members. These changes might obscure vocal individuality [42]. Given the reality of this variation, the greater the number of phrases used to calculate the functions, the more powerful the functions would likely be. In the other six situations in which the number of phrases used to calculate functions was larger or roughly equivalent to the number of phrases waiting for classification, the classification accuracy was 100%. After we increased the number of phrases used to calculate the discriminant functions, the classification accuracy of G4 male in 2012 also increased to 100%. Another reason might be that gibbons may change their vocalization during development, which has been documented in female gibbons [43]. Based on our own observations, subadult male cao vit gibbons produce relatively simple structured phrases when they begin to sing solo bouts that develop into complicated adult male phrases after intensive practice for 1–2 years.

As we demonstrate here, using discriminant function analysis to identify individual male gibbons by their unique vocalizations has multiple uses. Currently, the triangulation method developed by Brockelman and Srikosamatara [8] is widely used to survey gibbon populations [44]–[48]; however, when different groups in close proximity sing at different times, it can be difficult to obtain an accurate population estimate [47]. By combining triangulation with individual recognition using vocal individuality in male songs, we may be able to obtain more accurate estimates of population size, which are crucial for the conservation of small populations. We can also use vocal recording to monitor population dynamics in the cao vit gibbon population. These gibbons live in relatively stable territories and remain in the same territory after male replacement (unpublished data). If an adult male is replaced by a new male, DFA could be used to reliably tell them apart. Indeed, we observed one male replacement during our research, and it was confirmed using vocal identification. However, several practical issues should be considered before implementing this technique more broadly.

1. Gibbon songs should be recorded in the optimal season: Cao vit gibbons sing more regularly during the rainy season, especially in between July and September (unpublished data). We suggest recording gibbon songs during this period.
2. Record multiple bouts to identify individuals when possible: Paired males produce solo or duet bouts with females lasting on average 18.3 min [32], and adult males producing diverse phrases during each bout. While we have demonstrated that one bout is usually enough to identify an individual, two or more bouts are highly recommended if time and labor are available.
3. Gibbons should be recorded once per year: Male tenure is quite long in gibbons [49], and in this population, male tenure could last more than 6 years. As a result, recording male songs once every year should be enough to document male replacements, subadult male dispersals, and group loss. Although we were not able to test whether the vocalizations of subadult males remain stable after dispersal, Fan et al. reported that a western black crested gibbon (the closest relative of cao vit gibbons [50]) subadult male did not change its song after it replaced a neighboring group's male [22].
4. Cooperation between China and Vietnam is needed: Considering the sole remaining population of this species crosses the international border between China and Vietnam, it is difficult to implement any conservation or research activities without cooperation between these countries. Both subadult males and females disperse freely across the border [29] but people cannot. Vocal recording methods could be the only way to detect dispersal patterns uniformly in this transboundary population.

In conclusion, we have detected vocal individuality and stability in the critically endangered cao vit gibbons. Facilitated by their regular and loud morning songs, stable territories, and long-term male tenure, using vocal individuality to monitor gibbon population dynamics is feasible. Given the difficult terrain and international border that prevents tracking the dispersal of individuals in this area, the identification of vocal individuality could be the only method to detect dispersal patterns in this population. Furthermore, this time- and labor-saving method could be standardized easily in both China and Vietnam.
